# Prognostic factors of postoperative morbidity in surgery for resectable pancreatic cancer. Regional institute of neoplastic diseases ‘Dr. Luis Pinillos Ganoza’ IREN Norte. 2007–2022

**DOI:** 10.3332/ecancer.2024.1754

**Published:** 2024-09-05

**Authors:** Aldaír Guzmán-Aponte, Juan Alberto Díaz-Plasencia, Edgar Fermín Yan-Quiroz, José Richard Tenazoa-Villalobos

**Affiliations:** 1 School of Medicine, Universidad Privada Antenor Orrego, Trujillo 13008, Peru; 2 Instituto Regional de Enfermedades Neoplásicas, Trujillo 15036, Peru; 3 Hospital de Alta Complejidad Virgen de la Puerta – EsSalud, La Esperanza 13013, Perú; 4 Oncologic Surgery, Instituto Regional de Enfermedades Neoplásicas IREN Norte ‘Luis Pinillos Ganoza’, Trujillo 13001, Peru; 5 Victor Lazarte Echegaray Hospital – Essalud, Trujillo 13013, Peru; a https://orcid.org/0009-0002-3707-4137; b https://orcid.org/0000-0001-7019-6609; c https://orcid.org/0000-0002-9128-4760; d https://orcid.org/0000-0003-3622-9408

**Keywords:** type of pancreatic resection, resectable pancreatic cancer, nutritional index prognosis, postoperative morbidity

## Abstract

**Objective:**

To determine the prognostic factors associated with postoperative morbidity for resectable pancreatic cancer at the Instituto Regional de Enfermedades Neoplásicas del Norte ‘Dr. Luis Pinillos Ganoza’ - IREN Norte. 2007–2022.

**Materials and methods:**

A study was conducted with a case-based (22 patients) and control (14 patients) design nested in a cohort that included 36 patients who underwent proximal duodenopancreatectomy (Whipple) or distal pancreatectomy for pancreatic cancer.

**Results:**

In the present study, it was found that the total number of living patients represents 86.1% of the total (36 patients). Of the total population, patients who presented only 1 complication were 13.6% and more than 1 complication were 86.4%. It was also found that the most frequent complication in the general population was gastric emptying syndrome, which was present in 50% of all patients. In the bivariate analysis, a highly significant association was observed between the low prognostic nutritional index (*p* < 0.001, OD = 20.400, CI = 95%: (3.377–123.245)), the type of pancreatic resection (*p* < 0.001, OR = 52.500, CI = 95%: (5.174–532.669)) and postoperative morbidity. In contrast, no significant association was found between age ≥65 (*p* = 0.062), diabetes mellitus (*p* < 0.908), Wirsung diameter (*p* < 0.432), hospital stay (*p* < 0.075) and postoperative morbidity. In the multivariate analysis, serum total bilirubin level ≥20 μmol/L (*p* = 0.778), use of preoperative biliary drainage (*p* = 0.176), type of pancreatojejunal anastomosis (*p* = 0.533) and pancreaticogastric anastomosis (*p* = 0.504) were not statistically significantly associated with postoperative morbidity.

**Conclusion:**

The type of pancreatic resection and the nutritional prognostic index <40.5 are prognostic factors of postoperative morbidity in postoperative patients for resectable pancreatic cancer.

## Introduction

Pancreatic cancer is one of the most aggressive solid tumours with high mortality and morbidity rates worldwide [1]. In general terms, this type of cancer metastasizes by invading early, spreading first to the regional lymph nodes, then to the liver, and less frequently to the lung. Due to the few symptoms that appear before the disease progresses to an advanced stage, it has a poorer prognosis [2].

In both the US and Europe, pancreatic ductal adenocarcinoma is considered the fourth leading cause of cancer mortality, with a 5-year life expectancy of 8% in patients of all stages and a median overall survival of 3–5 months in unresectable cases [2, 3].

Life expectancy for a person diagnosed with pancreatic cancer can vary significantly depending on several factors, such as the stage of cancer at the time of diagnosis, the overall health of the patient and the response to treatment. In general, pancreatic cancer is known to be one of the most aggressive types of cancer and, unfortunately, is often diagnosed at an advanced stage. According to the American Cancer Society, the relative 5-year survival rates for pancreatic cancer are approx:

- (no signs of spread outside the pancreas): 44%.
- Regional (spread to nearby structures or lymph nodes): 15% (Pancreatic cancer survival rates) [4].

According to the Lima Metropolitan Cancer Registry, the incidence in Peru is 3.91 per 100,000 inhabitants [5]. In the city of Trujillo, the mortality rate is 4.5% [6]. In 2022, mortality rates in elderly women (AAPC: 1.76%; 95% CI: 0.36, 3.17) and men (AAPC: 2.25%; 95% CI: 0.68, 3.85) will increase [7].

Complete tumour resection has remained a central component of treatment since the middle of the 20th century. Despite improved results due to the evolution and progress in surgical techniques, with major pancreatic resections, only a few patients (10%–20%) have the opportunity to undergo this radical surgery, since when they do present, most show signs of advanced disease [8].

Among the various types of surgery frequently performed on patients with pancreatic cancer, numerous modifications of standard techniques have emerged in an attempt to reduce the risk of postoperative pancreatic fistula. So far, none of the techniques has proven to be significantly superior to others, therefore, the anastomosis is tailored according to the surgeon’s skills and experience, the type of pancreas and the characteristics of the pancreatic stump. Among the most commonly used anastomosis techniques, we have pancreaticojejunal or pancreaticogastric anastomosis [9, 10].

In a study conducted by Yoshifumi K. Head of the Department of Surgery at Mie University in Japan, they achieved a decrease in complications from 26.4% in the early periods of their surgery (1976 to 1993) to 11.3% (3 patients) in the latter period (1994–2000), 3% (3 patients) in the last period (1994–2000) and they proclaim that in the past periods, cholangitis, fistulas of pancreatic jejunal anastomosis and gastrointestinal bleeding, occurred in their first period, with 5.8% of fistulas in the first period and reduced to zero (0%). Of fistulas and complications in its late period, with anastomosis, duct to mucosa and placement of a stent support tube [11].

Before the procedure performed (anastomosis techniques), biliary drainage systems are used. These, such as internal-external biliary or external biliary drainage in which a biliary drainage catheter with a blocking loop and multiple lateral orifices or a catheter that ends in a biliary duct (not in the intestine), respectively, is used. Also, in other types of biliary drainage, one or several biliary drainage catheters are placed, external right-to-left, left-to-right, bilateral internal-external [12, 13]. Preoperative biliary drainage (PBD) may not be necessary in cases where early surgery is planned within 2 weeks. However, for patients with cholangitis, delayed surgery due to logistics, patients with high preoperative bilirubin levels (≥ 20 mg/dL) or the need for neoadjuvant chemotherapy, if indicated and at the same time associated with major postoperative complications [14, 15].

Shin *et al* [16] in a study of a total of 831 patients who underwent PBD and then underwent duodenopancreatectomy at 1–6 weeks, show that major complications occurred significantly more in the drainage group (*p* = 0.002). Multivariate analysis shows that major complications occur significantly in the 3 and 4-week interval (odds ratio, 1.863 and 2.523, respectively), whereas the early (1 to 2 weeks) or late (more than 4 weeks) periods have no association with complications.

Among the nonsurgical factors, preoperative serum total bilirubin levels are considered an independent variable affecting postoperative morbidity status at 30 days. The risk of a complicated postoperative course is 3 times higher for patients whose serum total bilirubin is ≥20 mg/dL [17, 18]. In a study by Billingsley *et al* [19] conducted between 1990 and 2000 in 123 medical centers, they demonstrate that the preoperative total serum bilirubin level is one of the significant predictors of postoperative outcome, such that levels above 20 mg/dL actually increase 30-day postoperative mortality.

These factors such as total serum bilirubin level may influence the prognosis of postoperative patients since progressive elevated bilirubin levels are associated with the proinflammatory state resulting from portal and systemic endotoxemia, as well as bacterial translocation, which may lead to induce an uncontrolled inflammatory cascade and develop complications. Since surgery in jaundiced patients has been considered to be accompanied by a higher mortality and morbidity rate, PBD was introduced to improve outcomes after surgery; likewise, it has been observed that biliary drainage may also have an influence due to the fact that bile contamination may occur, especially at the time of catheter placement. Over a prolonged period, there is an extensive inflammatory reaction to foreign bodies in the biliary tract that provides a suitable condition for bacterial colonization in the biliary tree leading to occlusion and eventually to a high risk of leakage of the anastomosis. after surgery. However, the quality of drainage, duration and method of drainage are implicated [17].

The most common postoperative complications are: postoperative pancreatic fistula, severe postoperative bleeding, biliary fistula, delayed gastric emptying syndrome, abdominal abscess, sepsis, surgical site infections and anastomotic leakage. These complications have a negative impact not only on patients’ quality of life and hospital costs, but also on long-term oncologic outcomes [20–25].

Mintziras *et al* [26] in their meta-analysis and systematic review study with a total of 7,604 patients, evaluate postoperative morbidity in patients undergoing pancreas surgery where 14 retrospective cohort studies were included; they show that the overall complication rate is 40.8% (*n* = 3,103 patients), postoperative complication rates range from 24.3% to 64%, severe complication rates (Clavien-Dindo ≥III) between 4.2% and 31%.

It is generally accepted in the various studies available that postoperative morbidity in pancreatic cancer is high, and over the last 20 years, numerous studies have attempted to determine the relationship between certain parameters and postoperative morbidity, the detection of which would make it possible to identify an adverse outcome of the disease and the subsequent death of the patient. This would allow the medical team to act effectively for the benefit of the patient to prevent or reduce the possibility of complications.

### Problem statement

What are the main prognostic factors associated with postoperative morbidity in surgery for resectable pancreatic cancer?

## Objectives

### General objective

- To identify prognostic factors associated with postoperative morbidity in surgery for resectable pancreatic cancer.

### Specific objectives

- To determine the association between preoperative serum total bilirubin level and postoperative morbidity in surgery for resectable pancreatic cancer of the pancreatic head.
- To determine the association between PBD catheter use and postoperative morbidity in surgery for resectable pancreatic cancer of the pancreatic head.
- To determine the association between low early postoperative prognostic nutritional index (PNI) and postoperative morbidity in surgery for resectable pancreatic cancer.
- To determine the association between the type of pancreaticogastric anastomosis and postoperative morbidity in surgery for resectable pancreatic cancer of the pancreatic head.
- To determine the association between the type of pancreaticojejunal anastomosis and postoperative morbidity in surgery for resectable pancreatic cancer of the pancreatic head.
- To determine the association between the type of pancreaticoduodenectomy (cephalic versus distal) and postoperative morbidity in surgery for resectable pancreatic cancer.
- To determine by multivariate analysis the factors associated with postoperative morbidity in pancreatic head pancreatic cancer.

## Material and methods

The present case-control, cohort-nested, observational, quantitative and analytical study analysed information from a consecutive series of 36 patients (census sample) with histopathological diagnosis of resectable pancreatic carcinoma attended at the Department of Surgery in Abdomen of the Regional Institute of Neoplastic Diseases of the North ‘Dr. Luis Pinillos Ganoza’- IREN north during the period from 2017 to December 2022, who meet the selection criteria.

### Selection criteria

**Inclusion criteria**

**Cases**

- Patients of both sexes, aged 18 years or older, postoperatively treated for resectable pancreatic cancer, who presented complications during the 30 postoperative days.

**Controls**

- Patients of both sexes, aged 18 years or older, postoperatively treated for resectable pancreatic cancer, who did not present complications during the 30 postoperative days.

**Exclusion criteria**

- Incomplete medical history.
- Postoperative patients with unresectable pancreatic cancer.
- Patients with corticotherapy.
- Patients with long-term renal insufficiency.
- Patients with long-term liver disease.
- Patients diagnosed with other types of cancers.
- Patients undergoing treatment with chemotherapy, radiotherapy or both.

## Operational definition of variables

### Dependent variable

#### Postoperative morbidity

These complications will be classified as: surgical site infection, postoperative pancreatic fistula, sepsis, severe postoperative bleeding, delayed gastric emptying and abdominal abscesses. The same are within the Dindo-Clavien classification in a grade ≥3 which is defined as: Complications that require surgical, radiological or endoscopic intervention with or without general anesthesia during the intervention. Complications occurring during hospitalisation up to 30 days will be considered [21, 23, 27–46].

#### Independent variable

- **Preoperative serum total serum bilirubin (TSB) level:** TSB taken before surgery [17, 19].
  Serum BT ≥20 μmol/L
- **Use of PBD catheter:** Use of drainage systems before surgery, such as internal-external biliary drainage or external biliary drainage in which a biliary drainage catheter is used [12, 13].
- **Type of anastomosis:** Different anastomosis techniques used after pancreatic cancer surgery. Pancreato-gastric and Pancreto-jejunal anastomoses were included, which represent any type of anastomosis between the pancreas and the stomach or jejunum respectively, according to the surgeon’s preference [10, 21].
- **Type of pancreatic resection:** Surgical procedure where the head, body and/or tail of the pancreas are usually removed. Procedure performed for proximal obstruction of the pancreatic duct not susceptible to endoscopic treatment, thrombosis of the splenic vein with left portal hypertension [47].
- **Nutritional prognostic index (NPI):** a simple and inexpensive marker calculated from serum albumin and total lymphocyte count [23, 48–50].
  ✓ **10 × serum albumin value (g/dL) + 0.005 × total lymphocyte count in peripheral blood**

#### Intervening variables

- Age: Time from birth to the time recorded in the patient’s medical history.
- Diabetes mellitus: It is considered when glucose levels are higher than 180 mg/dL or if HbA1C ≥6.5.
- Wirsung’s diameter: Also known as the main pancreatic duct, it is a parameter commonly evaluated in imaging. Its reported normal value varies according to age:
  - In people under 50 years of age, the normal diameter is between 1 and 3.5 mm.
  - In people aged 70–79 years, the normal diameter is 2 to 5 mm.
  In addition, abnormal dilatation of the pancreatic duct may indicate obstruction of the normal flow of pancreatic secretions due to a tumour or distal narrowing. This may occur in cases of acute pancreatitis, chronic pancreatitis or pancreatic neoplasms. Some studies suggest that pancreatic duct dilatation (≥2.5 mm) without obvious cause may be an independent predictor of pancreatic cancer.
- **Hospital stay:** days elapsed from admission to hospital after surgery until the corresponding discharge recorded in the medical record.

#### Data collection procedures

- For the implementation of the project, permission was requested and obtained from the Research Committee of the Human Medicine Studies Program of the UPAO and the Institute of Oncologic Diseases IREN Norte to access the archive area.
- Using the HIS system, the medical records of patients treated by abdominal surgery between January 2007 and December 2022 were searched, then filtered to obtain a list of medical records of postoperative patients with resectable pancreatic cancer who presented the prognostic factors and intervening variables considered.
- Taking into account the aforementioned inclusion and exclusion criteria, the clinical history of the patients was chosen in order to obtain similar characteristics in both groups. According to the procedure, all postoperative patients with complications within the first 30 days after surgery and recorded in their records were considered cases (Appendix 1). Each medical record was reviewed and randomly selected.
- All patients without complications in the first 30 days after surgery were considered as control. The data collection form (Annex 1) continued to be completed. In addition, the interventional variables were recorded, each medical history was reviewed and randomised until the necessary information was completed.

**Data processing, tabulation and presentation:** International Business Machine Statistical Package for Social Sciences V.26 software was used for data processing. The analysis of the results was carried out using statistics such as: frequency tables, cross tables, statistical graphs, chi-square tests considering statistical significance if the chance is <5% (*p* < 0.05): Continuity Correction, Fisher’s Exact Test and association measures such as odds ratio and of its corresponding 95% confidence interval (CI) and CIs. In addition, binary logistic regression analysis was applied in the multivariate analysis to determine the prognostic factors associated with postoperative morbidity in surgery for resectable pancreatic cancer.

### Ethical aspects

The study obtained permission from the Ethics and Research Committee of the Regional Institute of Neoplastic Diseases ‘Dr. Luis Pinillos Ganoza’- IREN Norte. In this study (cases and controls nested in a cohort); only the information of the patients was collected through their clinical records (clinical histories); taking into consideration the Helsinki declaration II). WMA [51] and the general health law N° 26842 (Declaration of Helsinki of the World Medical Association. Adopted by the 18th World Medical Assembly, Helsinki, Finland, June 1964 and amended by the 29th World Medical Assembly, Tokyo, Japan, October 1975, the 35th World Medical Assembly, Venice, Italy, October 1983 and the 41st World Medical Assembly, Hong Kong, September 2013) (General Health Law. NO. 26842. Concordances: D.S. Nº 007-98SA. Peru: 20 July 2013) [52].

## Results

### Baseline population

This study consisted of a sample of 36 clinical histories belonging to postoperative patients with resectable pancreatic cancer who met all the inclusion criteria.

### Living and dead patients, type of pancreatic resection and age group of the patients who participated in the study, IREN Norte, 2007–2022

We found that of the patients who died, the group of patients who presented complications versus the control group is 4 to 1. The highest number of patients in the case group corresponds to the age group less than or equal to 50 years old. Cephalic pancreaduodenectomy was performed in more than twice as many patients (Table 1).

### Complications of the patients who participated in the study, IREN Norte, 2007–2022

It was found that the ratio of the number of patients who presented only one complication to those who presented more than 2 was 1:6. It was also found that the complication that occurred in a higher percentage in these patients was gastric emptying syndrome and the one that occurred in a lower percentage was intra-abdominal abscess (Table 2).

### Characteristics of postoperative patients with resectable pancreatic cancer, IREN-North, 2007–2022

By bivariate analysis, the mean of the variables such as age, hospital stay, serum bilirubin level (patients operated on pancreatic head) and PNI value is obtained is: 54 ± 16 years, 13 ± 11 days, 10.91 ± 11.23 mg/dL and 40 ± 9.54, respectively. It was also found that the intervening variables of the present study such as age ≥65 years, Diabetes Mellitus and Wirsung’s Diameter, did not present association with postoperative morbidity in patients with resectable pancreatic cancer (Table 3).

### Prognostic factors associated with postoperative morbidity in surgery for resectable pancreatic cancer, IREN-North, 2007–2022

By means of the bivariate analysis, it was shown that there is a statistically significant relationship between the type of pancreatic resection (*p* < 0.001, OR = 52.500, CI = 95%: (5.174–532.669) and postoperative morbidity. Likewise, there is a highly significant statistical association (*p* < 0.001, OR = 20.400, CI = 3.377–123.245) between PNI (DAY 3) <40.5 and postoperative morbidity. Therefore, PNI (DIA 3) <40.5 and the type of pancreatic resection constitute a risk factor and are associated with postoperative morbidity, it also allows to understand that a patient submitted to cephalic pancreatoduodenectomy has approximately 51.500 times more risk of presenting morbidity and patients with PNI <40.5 present 19,400 times more risk of presenting postoperative morbidity in surgery for resectable pancreatic cancer in patients of the IREN-North, period 2007–2022 (Table 4).

### Prognostic factors associated with postoperative morbidity in surgery for resectable pancreatic head cancer, IREN-North, 2007–2022

Bivariate analysis also showed that serum bilirubin level ≥20 mg/dL (*p* = 1, OR = 0.706, CI = 95%: (0.057–8.700), Use of PBD (*p* = 0.234, OR = 0.235, CI = 95%: (0.025–2.215), Pancreatojejunal anastomosis (*p* = 1, OR = 0.75, CI = 95%: (0.088–6.388), Pancreato-gastric anastomosis (*p* = 1, OR = 0.909, CI = 95%: (0.107–7.718), do not present significant statistical relationship with postoperative morbidity in surgery for resectable pancreatic cancer (Table 5).

### Predictor variables in the logistic regression equation, for the data of the group of patients for surgery for resectable pancreatic head cancer, IREN Norte, 2007–2022

We evidenced that, by multivariate analysis logistic regression was applied using the method of Successive Backward Steps (Wald) considering all variables, the results of the significance of the predictor variables show that variables such as serum bilirubin level ≥20 mg/dL (*p* = 0.778, OR = 0.650, CI = 95%: (0.032–13.019), Use of PBD (*p* = 0.176, OR = 5.631, CI = 95%: (0.461–68.855), Pancreatojejunal anastomosis (*p* = 0.533, OR = 3.388, CI = 95%: (0.073–157.293), Pancreato-gastric anastomosis (*p* = 0.504, OR = 3.907, CI = 95%: (0.072–212.046), are not predictors of postoperative morbidity in surgery for resectable pancreatic cancer of the pancreatic head, in this study group. These variables were eliminated and logistic regression was applied again considering only the variables that contribute to the model. Then we again obtained that the use of PBD (*p* = 0.206, OR = 4.250, CI = 95%: (0.451–40.013) is not considered a predictor of postoperative morbidity in surgery for resectable pancreatic head cancer (Table 6).

## Discussion

Prognostic factors are crucial for decision making in pancreatic surgery to a large extent, because the optimal surgical technique and perioperative care after pancreatic resection are still debatable. Acinar cells are susceptible to ischemia and changes in the microcirculation in the pancreas attributed to surgical trauma can lead to acinar cell destruction. Tissue hypoxia likewise increases capillary permeability, leading to leakage of fluid and activated enzymes into the surrounding tissue and tissue necrosis [37].

In the present study, the average age of the patients was 54 ± 16 years and the majority of patients who presented morbidity corresponded to the age group ≤50 years, coinciding with the findings of Shahri *et al* [17] who recorded that within the group of patients who presented postoperative complications, 60% were between 40 and 65 years old and the average age was 54.90 ± 14.33 years. Also in the study by Jotheeswaran *et al* [50] in which the average age was 48 years. According to the bivariate analysis of the present study, age is not statistically significant (*p* = 0.062). These results are consistent with previous studies [15, 50, 53, 54] in which age ≥65 years and postoperative morbidity were not significantly associated. On the contrary, in two studies [16, 31] there is a statistically significant association (*p* < 0.001).

With respect to the bivariate analysis with the variable diabetes mellitus, in the present study it was not a predictor of postoperative morbidity. Coinciding with several studies [15, 29, 54, 55] in which diabetes mellitus was found to be a predictor of postoperative morbidity. On the contrary, Shin *et al* [16] in their study with a population of 1,568 patients who underwent duodenal pancreatectomy and El Asmar *et al* [56] with a population of 117 patients undergoing duodeno-pancreatectomy found a statistically significant association between diabetes mellitus and postoperative morbidity (*p* < 0.001, *p* = 0.039).

In the results of the bivariate analysis of the present study, no significant association was found between Wirsung diameter ≥3 mm and postoperative morbidity. In other studies [15, 23, 37, 50] found no significant association between Wirsung diameter <3 mm and postoperative complications.

In the present study, the average hospital stay was 13 days, which coincides with the average of the study by Granero [21]. Granero [21] and Shin *et al* [16] study, which was 13 and 14 days respectively, in the study by Nakeeb *et al* [15] and was 8 days. Additionally, in the study by Degisors *et al* [54] in his bivariate analysis does show a statistically significant association (*p* < 0.001) between hospital stay and postoperative morbidity.

When a tumour within or adjacent to the bile ducts impedes the normal passage of bile from the liver to the intestinal tract, malignant bile duct obstruction occurs. Tumours of pancreaticobiliary origin, such as cholangiocarcinoma and cancer of the gallbladder and pancreas, are the most common causes of malignant bile duct obstruction. Other etiologies include lymphoma and metastasis of any primary neoplasm. Obstruction of the biliary tree blocks the normal bile excretion pathway, which is associated with an inflammatory response and increased systemic and portal levels of endotoxins and different cytokines such as tumour necrosis factor and interleukin. This leads to measurable biochemical disturbances including elevation of serum bilirubin (conjugated hyperbilirubinemia), γ-glutamyl transpeptidase and alkaline phosphatase. Physical signs and symptoms are usually present and many are explained by the absence of bile in the intestinal tract or the appearance of bilirubin and bile salts in the serum. The presence of one or more of these signs and symptoms of obstructive jaundice usually prompts referral for biliary drainage. Progressively elevated bilirubin levels are associated with the proinflammatory state resulting from portal and systemic endotoxemia, as well as bacterial translocation, which can lead to the induction of an uncontrolled inflammatory cascade that develops complications [12, 13, 15, 17].

In the present study the mean value of total serum bilirubin level was 10.91 ± 11.23 mg/dL, in the bivariate (*p* = 1, OR = 0.706, CI = 0.057–8.700) and multivariate analysis (*p* = 0.778, OR = 0.650, CI = 0.032–13.019) did not present a statistically significant relationship, on the contrary Shahri *et al* [17]. In contrast, Shahri *et al* [17], in his retrospective observational study obtained in his bivariate analysis that total serum bilirubin level ≥20 mg/dL significantly increased morbidity after pancreatoduodenectomy almost 3 times (*p* = 0.012, Odd ratio = 3.04, 95% CI: 0.887–10.438) and concluded that in his multivariate analysis it was a predictor of morbidity status (*p* = 0.009). Similar studies such as that of Chen *et al* [18] found that bilirubin ≥13 mg/dL was significantly associated with the cumulative number of complications (*p* < 0.001), unlike the multivariate analysis, where a positive relationship was found between the increase in bilirubin ≥18 mg/dL and the cumulative number of complications.

Considering that surgery in jaundiced patients goes hand in hand with a higher mortality and morbidity rate, PBD was introduced to improve post-surgical outcomes. However, in a prolonged period (more than 4 weeks), an extensive inflammatory reaction to foreign bodies in the biliary tract may occur, which provides a suitable condition for bacterial colonization in the biliary tree, leading to occlusion, resulting in severe inflammation and thickening of the wall of the duct of Wirsung leading to increased risk of complications after surgery [15, 17].

In the present study, both in the bivariate analysis (*p* = 0.234, OR = 0.235, CI = 0.025–2.215) and in the multivariate analysis (*p* = 0.176, OR = 5.631, CI = 0.461–68.855), the use of PBD did not present a statistically significant relationship, coinciding with several studies by Shahri *et al* [17], Nishida *et al* [29] and Lale *et al* [55] which conclude in their bivariate analysis (*p* = 0.999; *p* = 0.261; *p* = 1; respectively) that the use of preoperative drainage is not a predictive factor for postoperative morbidity. In contrast to the bivariate analysis of the study by Nakeeb *et al* [15] which determined that preoperative drainage is statistically significant with complications such as Delay in gastric emptying (*p* = 0.005), Infection at the operative site (*p* = 0.004) as well as the study by Sandini *et al* [57] by bivariate analysis, the use of short duration preoperative drainage, led to a higher morbidity rate (43.4% versus 20.0% versus 24.2%; *p* < 0.001). In their multivariate adjusted model they identified that the use of short duration preoperative drainage is an independent risk factor for complications after pancreatoduodenectomy (OD: 2.64; 95% CI: 1.23–5.67; *p* = 0.013).

The postoperative PNI value may be associated with hypoalbuminemia because serum albumin level is an important parameter in PNI. Albumin is a negative acute phase protein that is synthesized in the liver at a level of 12 to 25 g/day and a half-life of 20 days. The serum albumin level is decreased by multiple factors including malignancy, inflammatory state, trauma and surgery. This leads to poor tissue healing, decreased collagen synthesis in surgical wounds or anastomoses and impaired immune responses, such as macrophage activation and granuloma formation [23].

In the present study, we found that the mean value of PNI <40.5 was 40 ± 9.54, in the bivariate analysis we obtained that there is a statistically very significant association (*p* < 0.001, OR = 20.400, 95% CI: (3.377–123.245). Partly coinciding with the study of Rungsakulkij *et al* [23] in which he found that the mean PNI was 40.5 in his bivariate analysis (*p* < 0.01, OD = 2.619, CI = 1.17–5.83) and in the multivariate analysis (*p* < 0.05, OD = 2.776, CI = 1.21–6.38) which concludes that PNI ≥40.5 is an independent risk factor for complications after pancreato-duodenectomy.

Pancreatoduodenectomy is one of the greatest challenges in gastrointestinal surgery, with <5% mortality in high volume centers and even 50% of perioperative complications. Pancreatic stump anastomosis is considered the most difficult phase of surgery, crucial for postoperative healing. Technical failure in this step leads to postoperative pancreatic fistula, a life-threatening complication [9].

In the study by Daniel *et al* [58] found that the type of anatomy performed, either pancreatojejunal or pancreatogastric, was not associated with postoperative morbidity (Postoperative pancreatic fistula).

In the present study it was found that, both in the bivariate (*p* = 1, OR = 0.750, CI = 0.088–6.388) and multivariate analysis (0.533, OR = 3.388, CI = 0.073–157.293), the type of pancreatojejunal anastomosis is not a prognostic factor for postoperative morbidity. Coinciding with the study by Lale *et al* [55] showed in their bivariate analysis (*p* = 0.135) that there is no statistically significant association between pancreatojejunal anastomosis and postoperative morbidity. In contrast to the study by di Mola *et al* [59] where the two studied variants of pancreatojejunal anastomosis are statistically significant with postoperative morbidity (*p* = 0.018). And multivariate logistic regression analysis showed that one variant is an independent predictor of postoperative complications: pancreatic fistula (OR = 24.58, 95% CI = 1.71–354.32, *p* = 0.019) and postoperative hemorrhage (OR = 12.71, 95% CI = 1.23–131.55, *p* = 0.033). In other studies [60–62] found that the pancreatic jejunal anastomotic technique appears to be an easy, time-saving and safe technique with favourable rates of clinically relevant postoperative pancreatic fistula.

In the present study, it was found in the bivariate analysis that pancreaticogastric anastomosis (*p* = 1, OR = 0.909, CI = 0.107–7.718), did not present a significant statistical relationship with postoperative morbidity and in the multivariate analysis (*p* = 0.504, OR = 3.907, CI = 0.072–212.046), it was not considered a predictor of postoperative morbidity. Contrary to the meta-analysis by Hallet *et al* [63] which found that pancreatogastrostomy reduced the risk of postoperative complications (pancreatic fistula) (relative risk0.41, 95% CI 0.21–0.62). In the study by Herrera *et al* [64] found that the feasibility of pancreatogastrostomy was 90.5%. One hundred and six (53%) patients had complications, 36 (18%) were severe (Clavien-Dindo grade ≥III) and Keck *et al* [65]. The rate of postoperative grade B and grade C pancreatic fistula after pancreaticogastric versus pancreatojejunal anastomosis was not different.

In the bivariate analysis of the present study (*p* < 0.001, OR = 52.500, CI = 5.174–532.669), a statistically significant relationship between type of pancreatic resection and postoperative morbidity is evident. Like Lermite *et al* [66] and Dominguez-Comesaña *et al* [67] in their articles mention that in their results distal pancreatectomy is associated with less morbidity and shorter hospital stay than duodenopancreatectomy. Also Kusakabe *et al* [68] both in his bivariate and multivariate analysis identified as an independent risk factor for morbidity (exocrine pancreatic insufficiency) the performance of duodeno-pancreatectomy (versus distal pancreatectomy) (*p* < 0.0001, *p* < 0.0001). In the study by Sánchez-Morales *et al* [69] in his bivariate analysis found that the 44 patients undergoing open and also laparoscopic distal pancreatectomy is not statistically significantly with postoperative complications such as postoperative pancreatic fistula (*p* = 0.36, *p* = 0.36).

Mulliri *et al* [70] in his study found that the prevalence of DGE varies according to the type of pancreatic resection. After duodenopancreatectomy, 10%–45% of patients present complications (such as delayed gastric emptying among others) and after distal pancreatectomy, 8% of patients present complications (such as delayed gastric emptying among others) and after distal pancreatectomy, 8% of patients present complications (such as delayed gastric emptying among others).

Endocrine tissue consists of clusters of cells that form the islets of Langerhans and are scattered throughout the pancreatic parenchyma. The endocrine parenchyma includes four major cell types: insulin-producing β-islet cells, glucagon-producing α-cells, pancreatic polypeptide-producing cells and somatostatin-producing ∂-cells. Islets are distributed twice as densely in the tail as in the head and body of the pancreas, which explains why long-term impairment of glucose metabolism may be a common sequela after distal pancreatectomy. Diabetes after pancreatoduodenectomy is caused by loss of cell mass in the cephalic islets of Langerhans that correlates with both the extent of parenchymal resection and the loss of the duodenum; indeed, the duodenum secretes gastrin inhibitory peptide and glucagon-like peptide, which are important for intestinal stimulation of insulin secretion. After distal pancreatectomy, which always includes a duodenoectomy, postprandial insulin release is reduced. Thus, pancreatoduodenectomy considerably weakens the entire insular axis. Pancreatic α-cells and pancreatic polypeptide cells are predominantly located in the pancreatic head, which explains the decrease in circulating levels of glucagon and pancreatic polypeptide measured after distal pancreatectomy and the consequent decrease in their anti-insulin effect and inhibition of hepatic gluconeogenesis. The resolution of postoperative complications such as diabetes observed in some patients after pancreatoduodenectomy may be explained in part by the decrease in glucagon and pancreatic polypeptide release measured [70].

## Conclusion

1. The most common postoperative complication in this study was gastric emptying syndrome.
2. Within the group of patients who presented complications, 14% presented only one complication and 86% presented two or more postoperative complications.
3. Bivariate analysis of intervening variables such as age, diabetes mellitus, Wirsung diameter and hospital stay found no association with postoperative morbidity in postoperative patients with resectable pancreatic cancer.
4. Type of pancreatic resection and NPI <40.5 are prognostic factors for postoperative morbidity in postoperative patients with resectable pancreatic cancer; with odds ratios of 52,500 and 20,400, which were significant (*p* < 0.001).
5. No association was found; by bivariate analysis, between Serum bilirubin level ≥20 mg/mL, Use of PBD, Pancreaticojejunal Anastomosis and Pancreatogastric Anastomosis with postoperative morbidity in patients postoperatively treated for resectable pancreatic head cancer.
6. In the multivariate analysis that serum total bilirubin level ≥20 mg/mL, the use of PBD, pancreaticojejunal anastomosis and pancreaticogastric anastomosis were not considered as prognostic factors for postoperative morbidity in postoperative patients with resectable pancreatic head cancer.

## Recommendations

1. It is useful to consider the results reported in this study to strengthen surveillance strategies for early and timely recognition of postoperative complications in patients pancreatectomized for resectable pancreatic cancer.
2. Considering the economic factor and accessibility. The usefulness of new epidemiological, surgical and non-surgical variables should be investigated. In order to predict the occurrence of postoperative complications in patients with pancreatectomized and diagnosed with resectable pancreatic cancer.
3. Further research on the disease and other regional, national institutes, hospitals nationwide is recommended. Adequately controlled prospective studies are also needed to address postoperative and long-term prognostic factors and to determine whether the results described in our work can be extrapolated to the entire medical population.
4. It is essential to evaluate the usefulness and relevance of the PNI and the type of pancreatic resection in terms of their predictive ability for different outcomes in the context of pancreatic cancer, such as in-hospital and out-of-hospital mortality.
5. To obtain results consistent with reality, new studies with larger sample sizes should be carried out in countries similar to ours.

## Limitations

1. As this was a case-control study, the possibility of disproportionate information arising exists due to the lack of medical records necessary for the variables to be correctly identified. This reduces quality and precision.
2. There was a serious deficiency of information in national studies that could support the comparison with the variables investigated in this study.
3. The most important limitations of our study are that the study design is retrospective in nature and the small study population.

## List of abbreviations

IREN-North, Regional Institute of Neoplastic Diseases; PBD, Preoperative biliary drainage; PNI, Predicted nutritional index; TSB, Total serum bilirubin.

## Conflicts of interest

Non-financial conflicts of interest in the publication of this article.

## Funding

The authors declare that they have no financial.

## Figures and Tables

**Table 1.** Number of living and dead patients, type of pancreatic resection and age group of patients who participated in the study, IREN Norte, 2007–2022.

	Cases	Controls	Total
Patients			
Living patients	18	13	31 (86.1%)
Deceased patients	4	1	5 (13.9%)
Type of pancreatic resection			
Cephalic	21	4	25 (69.4%)
Distal	1	10	11 (30.6%)
Age group			
≤ 50 years	7	8	15 (41.7%)
51–60 years	4	3	7 (19.4%)
61–70 years	6	3	9 (25%)
≥71 years	5	0	5 (13.9%)

IREN – Collection records – Medical records archive: 2007–2022

**Table 2.** Complications in patients who participated in the study, IREN Norte, 2007-2022.

Morbility	N° Patients	Percentage
Postoperative pancreatic fistula (POPF)		%
Yes	13	36.1
No	23	63.9
Sepsis		
Yes	14	38.9
No	22	61.1
Postoperative hemorrhage		
Yes	12	33.3
No	24	66.7
Gastric emptying syndrome		
Yes	18	50.0
No	18	50.0
Operative site infection		
Yes	8	22.2
No	28	77.8
Intra-abdominal abscess		
Yes	4	11.1
No	32	88.9
Complications		
1 complication	3	13.6
>1 complication	19	86.4

IREN – Collection records – Medical records archive: 2007–2022

POPF: Postoperative pancreatic fistula

**Table 3.** Characteristics of patients postoperatively treated for resectable pancreatic cancer, IREN-North, 2007–2022. Univariate analysis.

Factors	Cases *n* = 22	Controls *n* = 14	Total		
	Postoperative morbidity				
	Yes	No		*p*-value	OR (95% CI)
Age (years)	60 ± 12	45 ± 17	54 ± 16		
≥65	8 (36.4%)	1 (7.1%)	9 (25%)	0.062	7.429 (0.814–67.831)
<65	14 (63.6%)	13 (92.9%)	27 (75%)		
Diabetes mellitus					
HbA1C ≥6.5	13 (59.1%)	8 (57.1%)	21 (58.3%)	0.908	1.083 (0.279–4.210)
HbA1C <6.5	9 (40.9%)	6 (42.9%)	15 (41.7%)		
Wirsung diameter (mm)					
≥3 mm	7 (31.7%)	2 (14.3%)	9 (25%)	0.432	2.800 (0.489–16.036)
<3 mm	15 (68.2%)	12 (85.7%)	27 (75%)		
Hospital stay(days)	18+-12	6+-3	13 ± 11	0.075	--

IREN – Collection records – Medical records archive: 2007–2022

HbA1C: Glycosylated hemoglobin test

**Table 4.** Prognostic factors associated with postoperative morbidity in surgery for resectable pancreatic cancer, IREN-North, 2007–2022. (Bivariate analysis).

Factors	Cases *n* = 22	Controls *n* = 14	Total	Statistics			
	Postoperative morbidity			Chi-square tests	*p* value	Odds ratio	CI (95%)
	Yes	No					
Type of pancreatic resection				15.022	0.000**	52.500	(5.174–532.669)
Cephalic	21 (95.5%)	4 (28.6%)	25(69.4.%)				
Distal	1 (4.5%)	10 (71.4%)	11 (30.6%)				
PNI (Day 3)<40.5	35.47 ± 8.35	47.13 ± 6.54 40 ± 9.54		13.619	0.000**	20.400	(3.377–123.245)
<40.5	17 (77.3%)	2 (14.3%)	19(52.8%)				
40.5	5 (22.7%)	12 (85.7%)	17(47.2%)				

IREN – Collection records – Medical records archive: 2007–2022

PNI (DAY 3): Prognostic nutritional index at 3 days

*p < 0.05, significant

** *p* < 0.01, highly significant

**Table 5.** Prognostic factors associated with postoperative morbidity in surgery for resectable pancreatic head cancer, IREN-North, 2007–2022. (Bivariate analysis).

Factors	Cases *n* = 21	Controls *n* = 4	Total	Statistics			
	Postoperative morbidity			Chi Square	*p* value	Odds ratio	CI (95%)
	Yes	No					
Serum bilirubin level (mg/dL) 9.94 ± 10.06 15.76 ± 16.94 10.91 ± 11.23				0	1	0.706	(0.057–8.700)
Yes	4 (19.0%)	1 (25.0%)	5 (20.0%)				
No	17 (81.0%)	3 (75.0%)	20(80.0%)				
Use of PBD				0.476	0.234	0.235	(0.025–2.215)
Yes	4 (19.0%)	2 (50.0%)	6 (24.0%)				
No	17 (81.0%)	2 (50.0%)	19(76.0%)				
Whipple + Pancreaticojejunal anastomosis				0	1	0.75	(0.088–6.388)
Yes	9 (42.9%)	2 (50.0%)	11 (44.0%)				
No	12 (57.1%)	2 (50.0%)	14 (56.0%)				
Whipple + pancreaticogastric anastomosis				0	1	0.909	(0.107–7.718)
Yes	10 (47.6%)	2 (50.0%)	12 (48.0%)				
No	11 (52.4%)	2 (50.0%%)	13 (52.0%)				

IREN – Collection records – Medical records archive: 2007–2022

**Table 6.** Predictor variables in the logistic regression equation, for the data of the group of patients for surgery for resectable pancreatic head cancer, IREN Norte, 2007–2022. Multivariate analysis.

Variables in the equation					
		Sig.	Exp(B)	95% C.I. for EXP(B)	
				Inferior	Superior
Step 1^a^	Serum total bilirubin level >=20 μmol/L	0.778	0.650	0.032	13.019
	Use of preoperatory biliary drainage	0.176	5.631	0.461	68.855
	Whipple+pancreatoyeyunal	0.533	3.388	0.073	157.293
	Whipple+pancreatogastrics	0.504	3.907	0.072	212.046
	Constant	0.457	0.013		
Step 4^a^	Use of preoperatory biliary drainage	0.206	4.250	0.451	40.013
	Constant	0.689	0.471		

a Variables specified in step 1: Serum total bilirubin level ≥20 μmol/L, Use of PBD, Whipple + pancreaticojejunal, Whipple+pancreatogastrics

IREN – Collection records – Medical records archive: 2007–2022

## Appendix 1

### FICHA DE EVALUACIÓN

“FACTORES PRONÓSTICOS DE MORBILIDAD POSTOPERATORIA EN CIRUGÍA POR CÁNCER DE PÁNCREAS RESECABLE”

#### PROTOCOLO DE RECOLECCIÓN DE DATOS

Fecha: __________________________ N.º: _______________________

**1. DATOS GENERALES:**

** 1.1. Registro de historia clínica: _______________**

** 1.2. Edad (años): _______________**

** 1.3. Diabetes mellitus SI ( ) NO ( )**

** 1.4. Diámetro del Wirsung SI ( ) NO ( )**

** 1.5. Estancia Hospitalaria postquirúrgica (días): _______________**

**2. VARIABLE INDEPENDIENTE**

** 2.1. Nivel Sérico de Bilirrubina Preoperatoria SI ( ) NO ( )**

** ≥ 20 μmol/L**

** 2.2. Uso De Catéter De Drenaje Biliar Preoperatorio SI ( ) NO ( )**

** 2.3. Whipple + Anastomosis Pancreatoyeyunal SI ( ) NO ( )**

** 2.4. Whipple + Anastomosis Pancreatogástrica SI ( ) NO ( )**

** 2.5. PNI(día3)<40.5 SI ( ) NO ( )**

** 2.6. Tipo de resección pancreática SI ( ) NO ( )**

**3. VARIABLE DEPENDIENTE**

** 3.1. Morbilidad Postoperatoria. SI ( ) NO ( )**

** 3.2. Infección de sitio operatorio SI ( ) NO ( )**

** 3.3. Fístula pancreática postoperatoria (POPF) SI ( ) NO ( )**

** 3.4. Sepsis SI ( ) NO ( )**

** 3.5. Hemorragia Postoperatoria Severa SI ( ) NO ( )**

** 3.6. Retraso del vaciamiento gástrico SI ( ) NO ( )**

** 3.7. Absceso Abdominal SI ( ) NO ( ).**
